# 3D Bioprinting in Tissue Engineering for Medical Applications: The Classic and the Hybrid

**DOI:** 10.3390/polym12081717

**Published:** 2020-07-31

**Authors:** Zelong Xie, Ming Gao, Anderson O. Lobo, Thomas J. Webster

**Affiliations:** 1 Department of Chemical Engineering, Northeastern University, Boston, MA 02115, USA; xie.ze@northeastern.edu (Z.X.); gao.min@northeastern.edu (M.G.); 2 LIMAV–Interdisciplinary Laboratory for Advanced Materials, BioMatLab, UFPI–Federal University of Piauí, Teresina 64049-550, Brazil; lobo@ufpi.edu.br

**Keywords:** 3D printing, bioprinting, hybrid additive manufacturing, tissue engineering, tissue regeneration

## Abstract

Three-dimensional (3D) printing, as one of the most popular recent additive manufacturing processes, has shown strong potential for the fabrication of biostructures in the field of tissue engineering, most notably for bones, orthopedic tissues, and associated organs. Desirable biological, structural, and mechanical properties can be achieved for 3D-printed constructs with a proper selection of biomaterials and compatible bioprinting methods, possibly even while combining additive and conventional manufacturing (AM and CM) procedures. However, challenges remain in the need for improved printing resolution (especially at the nanometer level), speed, and biomaterial compatibilities, and a broader range of suitable 3D-printed materials. This review provides an overview of recent advances in the development of 3D bioprinting techniques, particularly new hybrid 3D bioprinting technologies for combining the strengths of both AM and CM, along with a comprehensive set of material selection principles, promising medical applications, and limitations and future prospects.

## 1. Background

Printing technologies have been in societies for thousands of years. With the invention of Woodblock printing before 220 A.D. in China [1] and the subsequent development of the printing press in 15th century Europe [2], printing technology has significantly improved the reproduction of text and images, which has further expedited the dissemination of information. Undoubtedly, printing technology has played a revolutionary role in shaping the global society in many ways, including in language, education, industry, religion, and politics [3]. This is especially true regarding the last few decades, with advanced printing technologies shifting from two-dimensional (2D) surface printing to the production of 3D structures by continuously adding layers of materials to form 3D shapes. This has presented new paths for the application of this additive manufacturing process from rapid prototyping and manufacturing in biomedical, aerospace, and architecture industries [4] to the fabrication of customized consumer products, such as mechanical parts, wearables, model replicas, and even 3D-printed food [5,6,7].

The concept of 3D printing was first described by David E. H. Jones back in 1974 [8]. It was then established by Hideo Kodama using photo-hardening thermoset polymers for fabricating 3D plastic models as the early additive manufacturing (AM) process in 1981 [9]. Later in 1986, a 3D printing methodology named “stereolithography” was brought to light by Charles W. Hull, wherein layers of materials were sequentially printed layer-by-layer and then cured to form solid structures by being placed under ultraviolet (UV) light [10]. A later application of this process made it possible to create sacrificial resin molds for the fabrication of 3D scaffolds using biological materials. This was followed by the development of biomaterial direct printing into 3D frameworks using solvent-free, aqueous-based systems, which enabled transplantation with or without seeded cells [11]. Recent progress in nanotechnology, cell biology, and materials science has made it possible for 3D bioprinting to be used as a method for improved tissue engineering, which presents tremendous potential for more future advancements in medicine [12].

In 3D bioprinting, small units of biomaterials, biochemicals, and living cells are positioned precisely with functional components to fabricate tissue-like 3D structures [13]. Compared to the conventional use of 3D printing to form cell-free scaffolds, 3D bioprinting requires different technical approaches to construct 3D structures with mechanical and biological properties suitable for the deposition of living cells and the restoration of tissue and organ function, including biomimicry, autonomous self-assembly, and mini-tissue building blocks. [3]. There are several advantages of 3D bioprinting over typical 3D printing, including accurate cell distribution, high-resolution cell deposition, scalability, and cost-effectiveness. However, challenges remain for the development and subsequent applications of 3D bioprinting to be widely adopted among many industries, including medicine. To name a few, the selection of printable biomaterials is severely limited, current printing techniques need to be improved for faster printing speeds and better scalability, and even higher printing resolution is desired to produce specific biological functions without compromising mechanical properties.

In recent years, there have been a number of excellent review articles focused on the development of 3D bioprinting. S. V. Murphy et al. composed a systematic article detailing nearly every aspect of 3D bioprinting, including 3D bioprinting principles, imaging and design, techniques, and material selection [3]. There are also articles discussing specifically the advances in 3D printing technologies and materials, such as the review by H. N. Chia et al., which also summarizes numerous recent examples of how traditional non-biological 3D printing techniques have been improved for better biocompatibility and the fabrication of biomaterials [14]. Other authors, such as W. Jamróz et al., have focused on the pharmaceutical and medical applications of 3D bioprinting, with extensive coverage from wound dressings and implants to 4D bioprinting and biorobotics [15]. However, there are very few reviews that illustrate 3D bioprinting from all sides, including techniques, materials, and applications with the addition of emerging hybrid 3D bioprinting technologies to provide researchers with a more comprehensive overview on the development in 3D bioprinting and possible new directions for innovation.

In this review, recent advances of 3D bioprinting for tissue engineering are presented. First, we introduce the main strategies of 3D printing (both non-biological and bioprinting) and their advantages and disadvantages. Specifically, we present hybrid AM techniques for 3D bioprinting applications in tissue regeneration. Next, criteria for printable biomaterials and cell sources for 3D bioprinting are covered. The medical applications of 3D bioprinting are then explored. Finally, we discuss current limitations of and future perspectives for 3D bioprinting.

## 2. 3D Printing Processes and Techniques

### 2.1. Introduction

The general 3D printing process includes only a few steps: (1) The generation of 3D computer-based models of architectures using computer-aided design (CAD) and computer-aided manufacturing (CAM) tools and mathematical modeling techniques [16] based on imaging data obtained from computed tomography (CT) scanning, X-rays, and magnetic resonance imaging (MRI). (2) The production of 2D cross-sectional images from 3D computer models using tomographic reconstruction. (3) The building of 3D structures via a computer-controlled layer-by-layer deposition process. (4) The post-construction modification to meet specific demands, such as surface treatment for nanoarchitectures [14]. The variation among 3D printing techniques could affect the design of 3D computer models during the reconstruction of 2D slices to 3D scaffolds. Therefore, the features and properties of 3D printing systems must be taken into consideration during the design process.

Depending on the materials and manufacturing processes, typical 3D printing techniques could be classified as non-biological 3D printing and 3D bioprinting. Non-biological 3D printing could be represented by fused deposition modeling (FDM), stereolithography (SLA), selective laser sintering (SLS), selective laser or electron beam melting (SLM or EBM), and laminated object manufacturing (LOM). There are three primary methods for 3D bioprinting: inkjet, laser-assisted, and extrusion bioprinting, the details of which are discussed in Section 2.3 in this review. Some of the bioprinting techniques can be used for non-biological purposes, however, specific properties of non-biological methods listed here have limited their biological 3D printing applications.

There is a new trend of integrating AM with CM for the benefit of both technologies. This combined manufacturing, namely, hybrid additive manufacturing, has been widely applied in metal manufacturing for products with complex spatial constructs and precise surface finishes. The current progress of applying hybrid AM in tissue engineering at a variety of integration levels is discussed here and represents a novel direction for 3D bioprinting in medicine.

### 2.2. Non-Biological 3D Printing

We categorize the following printing methodologies as non-biological printing, which does not mean they have zero potential for biomedical applications. Instead, with a proper selection of printing materials, these techniques could generate some biological structures with unique features. However, the ideal material is exceptionally scarce because this material should bear long-time exposure to a high processing temperature and have the right figurability to form structures with desirable mechanical properties while being completely biocompatible. Besides, the nature of some techniques carries inevitable cytotoxicity, and some necessary post-processing is challenging for bio-applications [14]. However, it is vital to learn the drawbacks of those non-biological methods and discuss some recent improvements in these techniques.

#### 2.2.1. Fused Deposition Modeling

The most commonly used method in AM is fused deposition modeling (FDM). In FDM, a continuous filament of thermoplastic material is heated at the nozzle and melted into a semi-liquid state to be extruded onto the building platform or the previously printed layers when the platform lowers vertically [17]. The process happens in a layer-by-layer fashion, and the layers are fused together after the deposition of each layer. The solidification of the printed 3D structure takes place at room temperature after that. It is possible to use multiple extrusion nozzles during FDM. In some cases, a second nozzle is used to deposit temporary supporting materials for cantilevers [18]; in other cases, hybrid scaffolds are created by multi-nozzle fused deposition using multi-component materials [19]. One prominent feature of FDM is its ability to print frameworks with excellent mechanical properties, such as high porosity with an adjustable air gap size in the same layer or between layers. The geometry and morphology of the structure can be precisely controlled by the horizontal movement of the extrusion nozzles (x-y direction) and the vertical movement of the platform (z direction) at the governor of the CAD data files. Other main process parameters, such as layer thickness, width, deposition speed, deposition temperature, raster angle, and orientation of filament, have been widely studied for their impact on the printed structure’s mechanical properties [20,21,22].

FDM has the benefits of low cost, fast fabrication speed, and easy process operations. However, in terms of bio-applications, FDM shows limitations of material selection with optimal thermal and rheological properties but a lack of biocompatibility; it is also challenging to utilize living cells or other temperature-sensitive biomaterials for printing due to the high processing temperatures involved [14]. In recent years, there have been more biocompatible materials adapted to FDM to create scaffolds. For example, a bony scaffold made of polylactic acid (PLA) and hydroxyapatite (HA) was fabricated with desirable mechanical and biological performance in various porosity shapes, and pore sizes [23]; bio-polyethylene (BioPE) bio-composites reinforced with thermomechanical pulp (TMP) fibers and maleic anhydride (MAPE) showed significantly enhanced tensile strength, as developed by Q. Tarrés et al. [24]. Despite the fact that there are only a few biomaterials that have been developed for use in FDM, the number of biocompatible FDM filaments still pales in comparison to the number of thermoplastics used in CM methods such as injection molding.

#### 2.2.2. Stereolithography

Dating back to 1986, stereolithography (SLA) was the first of its kind—a rapid prototyping process brought to the public attention. SLA uses UV light or electron beams to initiate a polymerization chain reaction on a photocurable resin or monomer solution layer. After the completion of the solidification of a layer, in most cases in a bottom-up approach, the platform attached to the cured layer is lowered vertically to allow another layer of uncured liquid resin to be spread over the top. The process then polymerizes the upmost layer until the 3D structure is complete in a layer-by-layer fashion. In the case of a bottom-up approach, light is projected onto the bottom of the resin vessel through a transparent plate to cure the first layer of the resin solution it is in contact with. The cured layer is then lifted and detached from the bottom of the vessel to fill fresh resin in between the solid layer and the transparent plate so the next layer can be solidified [25]. The removal of excess uncured resin happens after printing is completed. Furthermore, post-processing treatments such as heating or photo-curing in a UV oven are used to convert any unpolymerized parts within the structure and enhance mechanical strength [26]. Key kinetic parameters of the curing reaction, such as the intensity of the laser source, scanning speed, and exposure duration, are used to control the curing time and the thickness of the polymerized layer, which are crucial to the quality of the printed structure [27,28,29]. Additionally, UV absorbers and photo-initiators can be added to the resin to control the depth of polymerization [30,31,32].

SLA is well known for its ability to print complex shapes with internal architectures at extremely high resolutions. X. Zhang et al. reported a 1.2 μm resolution using a micro-stereolithography (μSL) apparatus [33]. It was later reported by C. Sun et al. that an ultra-fine micro-spring array with a diameter of 0.6 μm was obtained [34]. The disadvantages of SLA include its high cost, relatively slow printing speed, and limited material selection of biocompatible resins with suitable SLA processing properties. Possible cytotoxicity of the uncured resin and residual photo-initiator [35], and weak mechanical properties of printed scaffolds, are some other concerns for SLA medical and hard tissue engineering applications.

#### 2.2.3. Selective Laser Sintering and Electron Beam Melting

Selective laser sintering (SLS) and electron beam melting (EBM) are both powder bed fusion processes consisting of thin layers of evenly spread and tightly packed fine powders on a platform. The laser or electron beam is used to scan the power particle surface in 2D patterns controlled by CAD data files. The powder particles are heated above the glass transition temperature and fused together with neighboring powders through molecular diffusion. The powder bed platform is lowered after the completion of one layer, and a fresh layer of powder material is rolled across the top, fused, and bounded with the previous layers until the 3D structure is built. Any unbonded powder is removed after the part is complete, and the necessary post-process heat treatment is applied to achieve full density [36]. The feature resolution is determined by powder particle size distribution, laser intensity, focused laser beam diameter, scanning spacing, and speed [37]. EBM or selective laser melting (SLM) is more commonly used to melt down pure metal powders. Intense energy is used in EBM or SLM to completely fuse powder particles into one fully-dense, consolidated structure, which results in superior mechanical properties [38]. In the case of dealing with large metal or alloy components with low complexity, direct energy deposition (DED) is used with a far higher amount of energy for melting metals. DED is useful for repairing and retrofitting large manufactured parts in situations wherein the application of SLS or EBM is limited [4]. The main advantage of both SLS and EBM is quality—high fracture toughness and mechanical strength—which makes either perfect for directly creating metallic implants that promote bone ingrowth and regeneration. However, the complexity in consolidation and molecular diffusion during the sintering process has limited the library of materials and the final feature resolution of printing [39].

### 2.3. 3D Bioprinting

**Figure 1:**
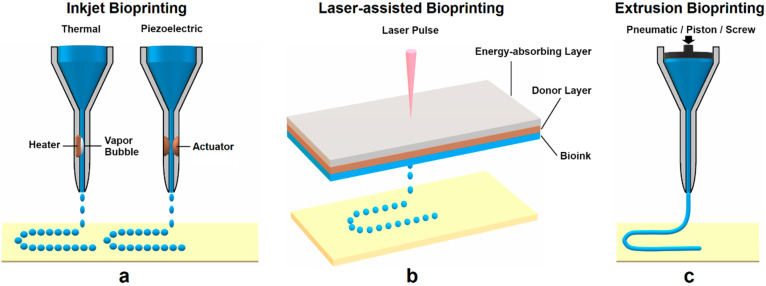
Common bioprinting techniques: (**a**) Inkjet bioprinting uses an electric heater or piezoelectric actuator to create a pressure pulse that propels the bioink droplet onto the substrates. (**b**) Laser-assisted bioprinting has a pulsed laser source and a ribbon structure (energy-absorbing layer, donor layer, and bioink layer). The laser pulse energizes the ribbon, generating a vapor bubble to propel bioink droplets onto the receiving substrate. (**c**) Extrusion bioprinting utilizes a pneumatic or piston or screw-based pressure to extrude the bioink through a micro-nozzle in the form of a continuous filament.

3D bioprinting has become an attractive method that allows the direct deposition of living cells while fabricating 3D complex constructs via a top-down approach rapidly. There are three main techniques use for the deposition and patterning biological materials: inkjet bioprinting, laser-assisted bioprinting, and extrusion bioprinting, as seen in Figure 1. A single bioprinting method cannot yet produce synthetic tissues and organs at all scales and complexities. All kinds of features of these techniques should be investigated in terms of crucial factors, such as printing resolution, cell viability, and the material to print desired 3D structures.

#### 2.3.1. Inkjet Bioprinting

Inkjet bioprinting, as the first bioprinting technique, is based on conventional inkjet printing processes [40]. Unlike common 2D inkjet practice, inkjet bioprinting uses a bioink (a hydrogel pre-polymer solution with encapsulated cells) as the material source. The bioink is ejected in the form of droplets onto the top of a substrate at a platform. The process is done in a continuous fashion to form patterns layer by layer, and the printed patterns solidify as the final 3D construct. The printer head uses either a thermal or piezoelectric force to generate drops with controllable sizes [13]. In thermal inkjet printers, the print head is heated electrically to press the droplets out of the nozzle. It has been demonstrated that the localized heating at the printer head for a short time only raises the overall temperature 4–10 °C [41]. This change of temperature does not impact the stability of biological molecules, such as DNA [42,43], or the viability or post-processing functions of mammalian cells [44,45]. The other type of inkjet printer contains a piezoelectric crystal inside the printer head. During the printing process, a voltage is applied to the crystal material, which causes a rapid change in shape to break the bioink into droplets from the nozzle at certain intervals [46]. The cell density in the bioink, the printing speed, and the nozzle size are some of the known factors that contribute to the resolution and mechanical properties of the inkjet-printed constructs. The advantages of inkjet bioprinting are its relatively fast printing speed, low cost, and easy accessibility. It is feasible to convert a commercially available printer to an inkjet bioprinter. N. D. Orloff et al. [47] reported a successful integration of a controller into the printer head of a modified HP G3110 scanner to build a bioprinter at a low cost. It was also shown by Z. Mohammadi et al. [48] that a modified HP Deskjet 1510 printer was capable of printing biological time-temperature indicators using a bioink. Despite these advantages, low droplet directionality, potential nozzle clogging due to high cell density, and the risk of exposure to thermal and mechanical stress during the droplet formation, are all concerns that apply to inkjet printers in bioprinting [49].

#### 2.3.2. Laser-Assisted Bioprinting

Laser-assisted bioprinting (LAB) is a derivative from direct-write [50,51] and laser-induced forward transfer techniques [52,53,54]. In LAB, the donor has a “ribbon” structure that consists of an energy-absorbing layer (e.g., titanium or gold) on the top and a layer of bioink (e.g., cells and hydrogel) at the bottom. In the printing process, focused laser pulses from the laser source stimulate an area at the energy-absorbing layer. Energy absorbed from this step vaporizes a portion of the donor layer to create a high-pressure bubble that propels the bioink onto the receiving substrate in the form of droplets. The quality of LAB-printed constructs is determined by many factors, such as the laser’s wavelength, intensity, and pulse time [55]; the surface tension, thickness, and viscosity of the bioink layer; the wettability of the substrate; and the air gap between the “ribbon” structure and the substrate [56].

Unlike other bioprinting methods, LAB is a nozzle-free and non-contact bioprinting procedure. LAB creates no mechanical stress towards the cells during printing, which results in the high viability of cells. Without a nozzle, LAB can print a wide range of biological materials with high viscosities, mammalian cells, and cells of high density without compromising cell viability and function [57,58,59]. It has a significant advantage over other bioprinting technologies, as the clogging of the nozzles can be avoided in LAB. On the other hand, preparing the “ribbon” setup for each cell or hydrogel type is time-consuming, mainly when multiple cell types are used or co-deposited with other materials. The side effects of the laser exposure onto the cells are still not known, nor fully understood [13]. The laser system operation is rather complex compared with nozzle-based printing, and precisely propelling cells is hard due to the nature of the “ribbon” cell coating [3].

#### 2.3.3. Extrusion Bioprinting

Extrusion printing has become one of the most economical techniques for rapid prototyping due to popular open-source projects such as Fab@home and RepRap [60]. Extrusion bioprinting could be seen as an extended application of inkjet bioprinting, the only one wherein extremely viscous materials and cells of high density can be deposited to form 3D structures. A continuous force, driven by a pneumatic pressure or piston or screw-based pressure, is used to extrude an uninterrupted line of bioink instead of liquid droplets via a micro-nozzle. The extruded material serves as a support structure after solidifying on the substrate; next, the platform is lowered horizontally and another layer of the bioink is added until the complete 3D construct is eventually formed. Compared to pneumatically-driven printers, the mechanical dispensing printers, including the piston and screw-based printers, provide more direct control over the material flow that leads to greater spatial control due to a delay of the compressed gas volume in pneumatic systems [61]. The viscosity and density of the bioink, the liquid phase of the bioink, the extrusion speed, and other material-specific properties, such as the capability of cross-linking between printed layers, are some of the main factors that need to be taken into consideration for achieving quality products from extrusion bioprinting.

One of the most important advantages of extrusion bioprinting is the ability to deposit a high-density of cells, enabling a more comprehensive range of material selection with a variety of cell densities and viscosities. With high-viscosity materials, extrusion bioprinting gains enhanced structural support with printed components, while with low-viscosity materials, a more suitable environment for maintaining cell viability and function can be created [3]. However, it has been reported that compared to inkjet-based bioprinting, extrusion bioprinting lacks a good strategy for preserving cell viability overall; the viability is typically in the range of 40–86%, with the rate diminishing with rising extrusion pressure and increasing nozzle gauge [62]. A comparison between non-biological 3D printing technologies and 3D bioprinting is summarized in Table 1.

### 2.4. Hybrid Manufacturing in Tissue Engineering

Although AM and CM are often placed on the opposite ends of the table, as manufacturing technologies advance, it is clear that a combination of both, known as hybrid manufacturing, could be more beneficial. The integration of AM and CM (also known as subtractive manufacturing) has come a long way. In metal manufacturing, typically, computer numerical control (CNC) machining for post-processing 3D-printed components is involved to deliver a smoother surface finish with greater accuracy. At a higher level of integration, the combining of additive and subtractive manufacturing processes within the same machine has been achieved for hybrid manufacturing. This combined hybrid manufacturing leverages the advantages of both technologies: the spatial complexity of AM and the high surface precision of subtractive approaches. At the same time, hybrid manufacturing accelerates the production process within a single operation [63]. For a detailed review of hybrid additive and subtractive machining, including the combination of CNC machining with arc or laser-based directed energy deposition, with additive cold spraying processes, powder bed fusion, or material jetting, the reader is directed to J.M. Flynn et al. [64].

**Figure 2:**
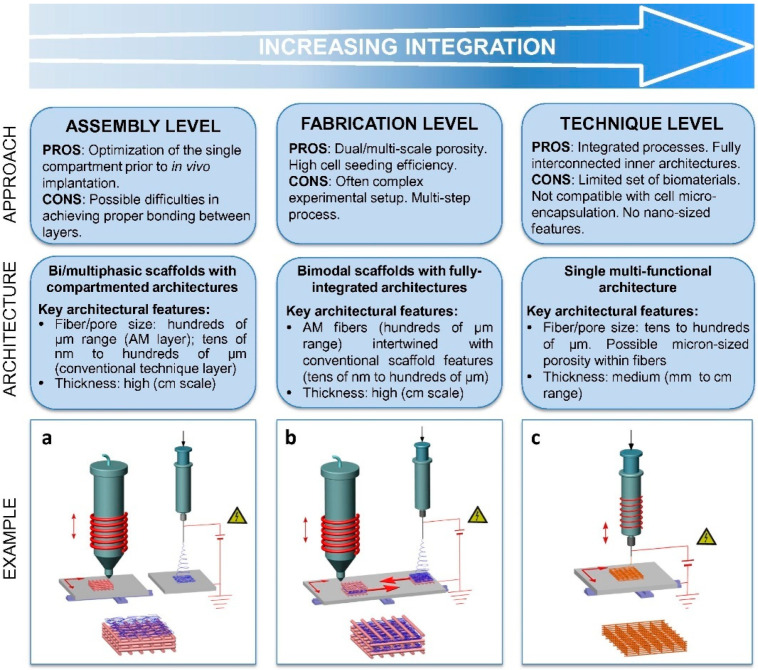
Classification of combined additive manufacturing techniques with details on advantages and limitations and key scaffold architectural characteristics for each approach. A combination of AM with electrospinning (ES) has been chosen as a representative example for the illustration of manufacturing equipment and obtained microstructures. In the drawings: (**a**) bonding between AM and ES scaffolds (assembly level); (**b**) deposition of AM and ES layers within a single scaffold (fabrication level); and (**c**) melt electrospinning direct writing as a de novo, single technique integrating the working principles of the two processes (technique level). Reproduced with permission from S. M. Giannitelli et al., Acta Biomaterialia; published by Elsevier, 2015 [67].

As for hybrid manufacturing in tissue engineering, specifically for the fabrication of scaffolds, the combination of AM with conventional scaffold fabrication techniques provides a new promising path forward. Even with recent efforts to enhance printing resolution, the relatively low resolution makes it unsuitable for AM to directly fabricate sub-micrometer structures that simulate the features of the natural extracellular matrix (ECM) or obtain hierarchical porous architectures with multimodal pore size distributions [65]. This inability could negatively affect cell adhesion and tissue regeneration. On the other hand, conventional fabrication methods, including solvent casting, particulate leaching, gas foaming, electrospinning (ES), phase separation, and freeze-drying, provide topographical tunability to achieve more vibrant features; however, they are limited regarding precise control of scaffold pore size, geometry, and interconnectivity [66]. To overcome these limitations, the hybrid combination between AM and CM is expected to generate new structures that can potentially satisfy the clinical demand for sophisticated tissue substitutes and meet requirements for functional tissue engineering constructs. Such structures should have control over scaffold microstructure, external shape, and pore size, allowing cell engraftment and migration, and adequate mechanical properties [67]. The integration of AM and CM can be implemented at several levels, as shown in Figure 2: by simply combining substructures made by AM and CM at the assembly level; by incorporating multi-length-scale frameworks into a single product at the fabrication level; and by fusing the principles of different fabrication techniques within a single, novel hybrid AM technology at the technique level.

#### 2.4.1. Hierarchical Integration of Modular Units at the Assembly Level

Multiphasic scaffolds, which refer to scaffolds containing two or more areas with different topologies, have been fabricated to achieve complicated and multiple tissues with functional interfaces via the hierarchical assembly of modular units. These scaffolds are made with various material types, internal structures (e.g., porosity, pore interconnectivity, etc.), cells, and biological parameters. In most cases, more than one fabrication technology is involved in this process [68,69]. Multiphasic scaffolds usually consist of an AM-fabricated solid compartment, and a soft phase, mostly represented by polymeric foams or textile meshes [70,71,72].

**Figure 3:**
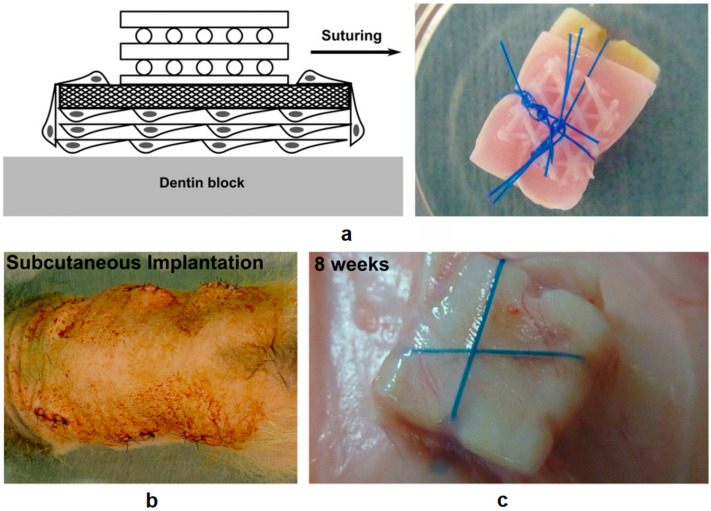
(**a**) Demonstration of the assembling a biphasic scaffold onto a dentin block. The blue cords are surgical sutures used to fix scaffolds together with the dentin block. (**b**) Illustration of subcutaneous implantation in athymic rats. (**c**) The tissue integration results after 8 weeks after implantation showed good mechanical stability of the construct with no signs of detachment. Reproduced with permission from C. Vaquette et al., Biomaterials; published by Elsevier, 2012 [71].

Electrospinning exemplifies one of the most common techniques for soft compartment production due to the similarity of electrospun matrices with the native ECM. It has been reported that Vaquette et al. employed an AM scaffold as the bone compartment and an ES membrane as the periodontal part to manufacture biphasic scaffolds for the regeneration of an alveolar bone/periodontal ligament complex. In this construct, the ES membrane serves as a supporting phase, promoting the adhesion of a periodontal ligament fibroblast cell sheet, and at the same time, the AM scaffold allowed for the spatial sustenance for bone restoration and provided biomechanical stability [71]. As seen in Figure 3, the biphasic scaffold/cell constructs were subcutaneously implanted into an athymic rat model to demonstrate the simultaneous regeneration of alveolar bone and periodontal ligament, and the formation of cementogenesis and periodontal attachment in vivo. The scaffolds showed good tissue integration following the implantation with no foreign body reaction or infection. No detachment of the biphasic scaffold from the dentin block it was attached to was observed during the implantation, proving high mechanical stability of the construct. In other work by H. Saniei et al., a screw-shaped bioabsorbable PLA implant was fabricated by FDM with a smooth surface. The surface of the screw was then coated with poly(vinyl alcohol) (PVA)-nano hydroxyapatite (nHA) nanofibers with various concentrations of nHA prepared by ES. All samples coated with PVA-nHA showed no cytotoxicity towards MC3T3-E1 cells seeded at the surface, and the sample P10-nHA1 (10 wt% with 1 wt% nHA) demonstrated the best cell proliferation performance and the highest cell viability (over 90%) on day 3 and day 7 of cell culturing [73]. This combination of FDM and ES fabrication showed a marvelous prospect for surface modification of 3D bioprinted scaffolds.

#### 2.4.2. Multi-feature Integration at the Fabrication Level

**Figure 4:**
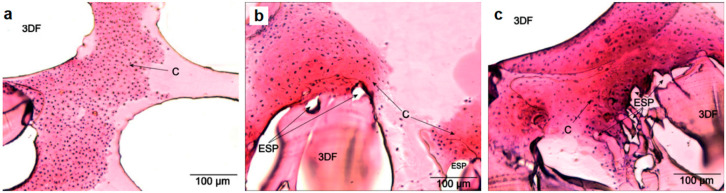
Histological cross-sections show sulphated GAG formation in (**a**) 3DF, (**b**) 3DFESP-30, and (**c**) 3DFESP-2 scaffolds by safranin-O staining. C indicates cartilage formation (GAG), 3DF refers to the macro fibers, and ESP refers to the microfibers. Reproduced with permission from C. A. van Blitterswijk et al., Advanced Functional Materials; published by John Wiley and Sons, 2007 [74].

Cell seeding efficiency is a crucial factor for optimal tissue regeneration; however, it is limited by the relatively low resolution of AM. To address this issue, a secondary submicrometer-scale material produced by CM can be incorporated inside the AM structure to simulate the hierarchical construction of natural tissues. This fabrication level integration of AM and CM approaches enables the AM compartment for stable support, while the superimposed microenvironment creates extra sites for better cell adhesion, and possibly provides distinct biochemical signals to guide cell behavior. L. Moroni et al. first successfully integrated electrospun matrices into AM scaffolds in a layer-by-layer fashion, resulting in improved biological activities, such as higher cell entrapment, proliferation, and more ECM production [74]. The enhanced tissue formation was indicated by measuring glycosaminoglycans (GAG), where the GAG amount increased from 160.29 ± 46.43 μg for the 3D fiber deposition (3DF) scaffolds (samples prepared by extrusion-based 3D bioprinting of macrofibers) to 321.1 ± 77.86 μg for the 3D fiber deposition and electrospun (3DFESP-30) scaffolds (samples prepared by electrospinning microfiber networks for 30 s between every two 3D fiber deposited layers) and to 316.84 ± 75.93 μg for the 3DFESP-2 scaffolds (samples prepared by electrospinning microfiber networks for 2 min between every two 3D fiber deposited layers) after a month. The formation of cartilage (represented by GAG) for the three different scaffolds can be seen in Figure 4. In a similar study reported by D. Sooriyaarachchi et al., a hybrid scaffold was fabricated by embedding electrospun aligned polycaprolactone (PCL) nanofibers between FDM prepared PLA frames. The resulting biomimetic scaffold exhibited a micrometer scale porous structure with enhanced cell attachment performance, well directed and organized cell growth and morphology, and enhanced mechanical properties [75]. It has also been reported that more native-like microenvironments have been integrated inside AM fabricated constructions via a conventional freeze-drying method [76] or unconventional layer-by-layer electrostatic self-assembly (E-LbL) [77]. In other cases, smaller AM-crafted structures were introduced into soft scaffolds as customized structural supports [78] or to enhance the overall biomechanical properties in the final product [79].

#### 2.4.3. Hybrid Additive Manufacturing at the Technique Level

Current AM technologies have been modified for designing biomimetic scaffolds with a more sophisticated level of AM and CM integration. However, most AM methods are not compatible with local-pore fabrication processes or standard porogen leaching processes. Therefore, efforts have been made by researchers to develop novel, AM-compatible porogen systems [80], and the use of indirect AM techniques [81,82,83].

**Figure 5:**
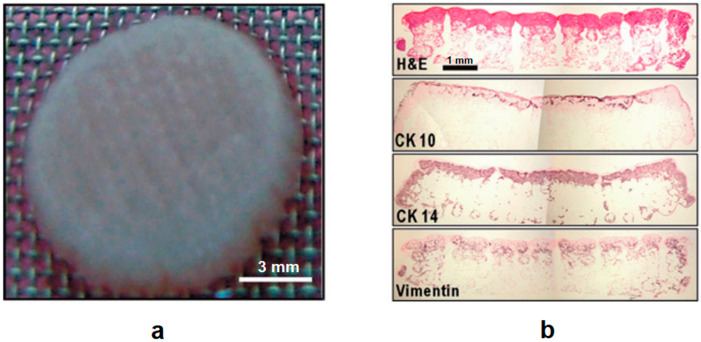
(**a**) Optical image of a collagen scaffold (diameter, 8 mm; thickness, 2 mm) after cell culturing with keratinocytes and fibroblasts; and (**b**) cross-sections with hematoxylin and eosin staining (top) and immunohistochemical detection of cytokeratin (CK10) and (CK14) (middle), and vimentin (bottom). Reproduced with permission from G. H. Kim et al., Journal of Materials Chemistry; published by Royal Society of Chemistry, 2009 [88].

Freeze drying methods have been integrated with standard AM techniques to combine submillimeter and micrometer-sized pores concurrently within a single 3D structure, which enables a greater surface area for cell adhesion and proliferation [84]. In this approach, a polymer solution is dispensed layer-by-layer at a low temperature; the deposited biomaterials are frozen and then lyophilized to remove the solvent to achieve various surface topologies [85]. An exemplary application reported by H. Yen et al. showed that poly(lactic-co-glycolic acid) (PLGA) scaffolds with different surface topographies on piling fibers were obtained by extruding PLGA solutions of different concentrations via liquid-frozen deposition manufacturing (LFDM) [86]. Other reported applications based on the same principle include low-temperature deposition manufacturing (LDM) [87], cryogenic direct-plotting [88], and rapid freeze prototyping (RFP) [89,90]. In the case of cryogenic direct-plotting, 3D collagen scaffolds were directly plotted using the 3D printing system coupled with a cryogenic system. The final printed scaffold was remarkably porous (>96%) and was 12% less than initially designed in size. The performance in terms of cell migration and differentiation was examined after two weeks of keratinocyte/fibroblast co-culture in the scaffold. As shown in Figure 5 the cross-sections of the scaffold were prepared after staining with hematoxylin and eosin, and after immunohistochemical staining with antibodies against cytokeratin (CK-10) and (CK-14), and vimentin. Results showed that cells readily migrated into and differentiated in the scaffolds (from the bottom to the surface of the scaffold) due to the scaffold’s well-designed pore structure [88].

Besides freeze-drying, 3D scaffolds with porous inner microstructures have been fabricated by a novel computer-assisted wet-spinning (CAWS) system. In this process, polymer filaments were deposited and solidified within a condensation bath with predefined layer-by-layer patterns. The resulting structures revealed a ‘‘spongy” morphology caused by a phase inversion process. As a result, enhanced biological responses were observed [91,92]. Other modifications of AM techniques include a melt electrospinning direct writing process [93,94] enhanced by the addition of a fast-motion automated collecting system [95,96], and an improved electrohydrodynamic (EHD) jet printing technique which enabled the fabrication of a 3D structure with high resolution below 10 μm [97] and desired filament orientation at room temperature [98].

## 3. Materials for 3D Bioprinting

In the first place, 3D printing was introduced for non-biological applications, such as the deposition of metals, ceramics, and thermoplastics. The involvement of a high processing temperature, the use of organic solvents, and the use of crosslinking agents are not compatible with living cells and biomaterials [3]. Therefore, finding biological materials that are compatible with the printing process and can also meet the mechanical and functional requirements for tissue constructs remains the main focus. Here, we firstly discuss the desired characteristics of ideal materials, followed by exemplary biomaterials. The critical factors for proper cell selection for 3D bioprinting are summarized at the end.

### 3.1. Material Characteristics

#### 3.1.1. Printability

One of the most important properties for a material to be suitable for 3D bioprinting is its ability to be well utilized by the printer—specifically, how well the material could be accurately deposited with the desired controllability. It is hard to define what printability is because it varies from one printing technique to another. For example, inkjet printing has a limitation for material viscosity, whereas extrusion-based printing can print materials with very high viscosity, but the latter requires the material to have a specific inter-layer crosslinking mechanism or shear-thinning properties. Since some processes involve high localized heating of the material for cell deposition, it is critical for the printing material or process to protect the cells from this high temperature. It has been found that materials with low thermal conductivity [99] or the ability to cushion the cells during the deposition process are more likely to result in increased cell viability and biological function after printing [100]. Another factor that has a significant impact on cell attachment and development is the surface tension between the printing material and the receiving substrate [101,102]. The printed material is expected to maintain vertical tension with the substrate. This can be achieved by coating the substrate with a thin layer of material to enhance its hydrophobicity before printing [103,104].

#### 3.1.2. Biocompatibility and Control of Degradation and Byproducts

Biocompatibility is described as the ability of a material to perform with an appropriate host response in a specific situation [105]. Over the years, the general goal of achieving biocompatibility has changed from requiring the implantation material to coexist with the host without causing any undesirable local or global effects to allow or actively produce desirable effects in the host passively [106]. Moreover, after the material is implanted into the host and degrades, it is expected that the material allows the cells to replace the material with its own produced ECM proteins at a speed that matches the degradation rate of the material in an ideal situation [107]. The generation of byproducts during the degradation process also defines the biocompatibility of the material as all byproducts should be nontoxic, readily metabolized, and rapidly cleared from the body.

#### 3.1.3. Mechanical Properties

Having sufficient structural and mechanical properties is crucial for the material to maintain a 3D structure after the solidification process. A stable structure is also essential for cells to attach, proliferate, and differentiate within a suitable environment [108,109]. It has been reported that the interactions between cells and the printing material affect cell adhesion significantly [110]. The mechanical requirements for materials are different for various types of tissue engineering, from hard implanted bone to soft tissues such as skin and cartilage; the mechanical properties are extremely critical as the functions of soft tissues mainly rely on such properties [111].

### 3.2. Biomaterials

Materials currently used in 3D bioprinting are either based on natural polymers (including collagen, gelatin, laminin, fibronectin, alginate, chitosan, fibrin, and hyaluronic acid (HA), often isolated from animal or human tissues) or synthetic polymers [112,113,114].

**Figure 6:**
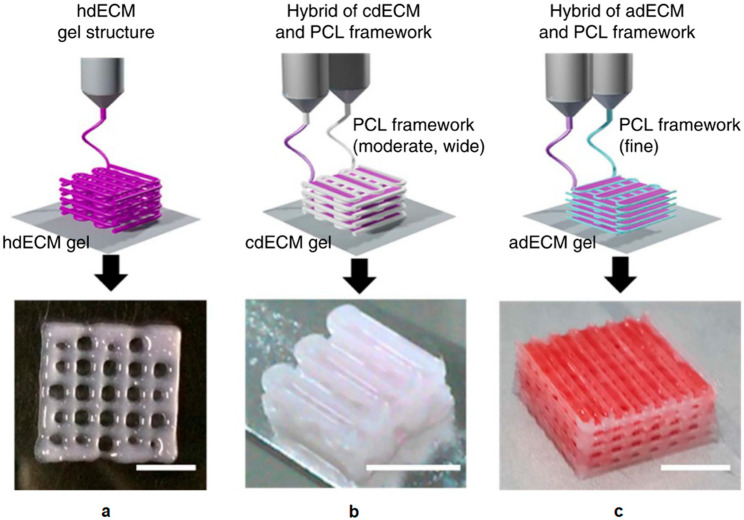
(**a**) Bioprinted heart tissue with only the heart dECM (hdECM). (**b**) Printed cartilage tissue with cartilage dECM (cdECM) and PCL framework. (**c**) Printed adipose tissue with adipose dECM (adECM) and PCL framework (scale bar, 5 mm). Reproduced with open access from F. Pati et al., Nature Communications; published by Nature Publishing Group, 2014 [116].

The advantages of using natural polymers are their similarities to native ECM and inherent bioactivities. The interactions between natural polymers and cells have been well established [115]. In recent years, the advances in decellularization of extracellular matrices make it a promising approach with which to obtain intact decellularized extracellular matrices (dECM) and incorporate them into bioprinting. F. Pati et al. reported the successful printing of bioinks containing dECM from three tissues [116]. The dECM compositions within the bioink carry various characteristics and biological functions from different tissues and could potentially closely resemble natural tissues. Examples of bioprinted constructs using three dECMs are shown in Figure 6.

On the other hand, synthetic polymers are made through chemical synthesis and can be finely tuned with specific chemical and mechanical properties to fit different bioprinting applications. Pluronics are ABA-type triblock copolymers, where the A block is hydrophilic polyethylene glycol (PEG), and the B block is hydrophobic polypropylene glycol (PPG). The advantageous gelation temperature and outstanding printability make Pluronics suitable for 3D printing [117]; however, as synthetic polymers, they show no bioactivity and are not intended for long-term cell viability maintenance [118]. Pluronics are often used as a sacrificial layer in 3D bioprinting instead [119]. PEG is also widely used in many compositions for 3D bioprinting, either to fabricate hydrogels or to create crosslinkable polymers after functionalization with diacrylate or dimethacrylate groups [117]. Poly (N-isopropylacrylamide) (PNIPAAM) is another type of synthetic polymer used in 3D bioprinting with a low solidification temperature of 30–37 °C. PNIPAAM is often combined with other natural polymers such as HA or alginate to improve its biocompatibility. M. Kesti et al. reported that a combination of HA-PNIPAAM with methacrylated HA (HAMA) resulted in excellent resolution of 3D-printed scaffolds with high viability of bovine chondrocytes of 80% after 3 h and 94% after 4 days post-printing [120].

### 3.3. Cell Sources

The selection of cells is critical during the fabrication of tissues and organs via 3D bioprinting. Printed tissues or organs should contain multiple types of deposited cells with specific and essential biological functions that best represent the native tissue or allow the stem cells to proliferate and differentiate into required cell types after the printing process [3]. Cells chosen for 3D bioprinting should accurately simulate the physiological states of cells in vivo and are supposed to maintain their in vivo functions under optimized microenvironments [121].

The accurate control of cell proliferation for desirable yet sufficient numbers of cells in vitro and in vivo is key for the 3D bioprinting of cells. Additionally, the manipulation of the cell proliferation rate is equally important. At the beginning stage, for populating the printed construct, a high proliferation rate is desired. Over time, the cell proliferation rate is expected to maintain tissue homeostasis without hyperplasia. Viral transfection [122] or the use of small molecules [123,124] is introduced to induce cell proliferation and prevent senescence.

For clinical purposes, cells for 3D bioprinting would be isolated from the patients to avoid potential negative immune responses in ideal situations [125]. However, the finite lifespan and the difficulty of isolating and culturing many primary cell types limit their application for bioprinting long-term tissue structures [126]. On the other hand, stem cells (such as stem cells from bone marrow [127] and fat [128] and perinatal stem cells from amniotic fluid [129]) are capable of proliferating and differentiating into specific cell types are considered promising for autologous applications.

Moreover, considering possible disadvantageous conditions during 3D bioprinting, including physical forces such as pressure and shear stress from inkjet or extrusion-based bioprinting, and potential laser exposure from laser-assisted bioprinting, it is necessary for the selected cells to be robust enough to survive these bioprinting processes. Inversely, to broaden the selection range of cell sources, it is also crucial for bioprinting technologies to be adaptable enough to incorporate cell types that are more sensitive to harsh processing conditions. A summary of biomaterials and cell sources mentioned above can be found in Table 2.

## 4. 3D Bioprinting for Medical Applications

3D bioprinting is rapidly expanding into a massive industry due to its diversity and potential applications. In general, the applications of bioprinting are categorized into two major groups: (1) tissue regeneration, such as the printing of blood vessels, heart valves, musculoskeletal tissues, liver, nerves, and skin; and (2) biomedical applications, including drug discovery and drug screening [130]. These are described more below.

### 4.1. Vessel and Heart Valve Applications

The vasculature plays a role in the transportation of nutrition and metabolic waste, which is a crucial factor for curing cardiovascular diseases [131] and the fabrication of tissues and organs with plentiful blood supplies. Although significant progress has been made for bioprinting of the vasculature in vitro, it remains challenging to achieve specific vascular features for different tissues. A vascular network bioprinted with methacrylated gelatin (GelMA) has been reported by L. Bertassoni et al. for its improvement in metabolic transportation, cellular viability, and endothelial monolayer formation [132]. For direct incorporation of the reduced size of vascular channels into bioprinted tissues, D. Kolesky et al. reported using the sacrificial bioink of Pluronic F127, which was later liquefied and removed at a lower temperature to obtain open vascular channels as small as 45 μm [133].

For bioprinting of heart valves, specifically aortic valves, extensive research has been done for printing with hydrogels. B. Duan et al. reported the development of the tri-leaflet heart valve conduit consisting of HA, gelatin, and human aortic valve interstitial cells, where high cell viability was observed after 7 days [134]. However, bioink materials are insufficient in terms of flexibility and elasticity, and their mechanical properties still do not meet clinical requirements [135]. Moreover, E. Chen et al. introduced a hybrid approach to fabricate tri-leaflet heart valve scaffolds by combing FDM and ES. Specifically, a PCL heart valve ring was first made as the mold by FDM, and then electrospun aligned nanofibers were cut and glued onto the ring to form the tissue engineering heart valve scaffold [78]. The resulting scaffolds showed good fiber alignment and high anisotropic mechanical properties.

### 4.2. Bone and Cartilage Applications

The engineering of artificial bone manufacturing is a common ground for both non-biological 3D printing and 3D bioprinting, and CM, including gas foaming [136,137], salt leaching [138,139], and dry freezing [140,141]. Among all available fabrication technologies, bioprinting has the unique advantage of the precise control of biological architectures and mechanical properties. Cement powder was used to fabricate biphasic calcium phosphate (BCPs) scaffolds containing hydroxyapatite and tricalcium phosphate (TCP) as the ideal composition for the repair and replacement of significant bone defects. The achieved structural accuracy of the BCPs scaffolds was higher than 96.5% [142]. F. Pati et al. reported the use of mineralized ECM generated by human nasal inferior turbinate tissue-derived mesenchymal stromal cells (hTMSCs) to ornament 3D bioprinted scaffolds containing PCL, PLGA, and b-tricalcium phosphate (b-TCP). After decellularization, the ECM-ornamented scaffolds enhanced both osteoinductive and osteoconductive properties with preserved organic and inorganic components [143]. Additionally, enhanced cell attachment and proliferation of human fibroblasts, osteoblasts, and bone marrow mesenchymal stem cells (MSCs) were reported by M. Wang et al. by the surface modification of 3D-printed PLA scaffolds with cold atmospheric plasma (CAP). It was shown that under optimal CAP conditions, both hydrophilicity and nano-scale roughness played a significant role in enhancing the printed constructs’ biological properties for bone regeneration [144].

The fabrication of cartilaginous tissues is another research-focused area for tissue engineering. J.-S. Lee et al. reported 3D bioprinted constructs consisting of PCL and cell-laden hydrogels with a PEG sacrificial layer for structural support. The porous material mixture was shown to be suitable for ear tissue regeneration with the occurrence of chondrogenesis and adipogenesis [145]. A newly formulated HA and alginate-based bioink were reported by C. Antich et al. for articular cartilage regeneration [146]. This bioink showed improved cell functionality (over 85% preserved cell viability after printing and increased GAG amount from around 23 μg/mL to 41.37 μg/mL after one month in culture) and promising 3D printability, mechanical properties, and biodegradability. C. Li et al. demonstrated a controllable fabrication of covalent hydrogels consisting of hydroxybutyl chitosan (HBC) and oxidized chondroitin sulfate (OCS) for cartilage repair applications. In this study, the hydrogels were injected into 3D extrusion bioprinted Pluronic F127 sacrificial modules to obtain designed inner structures and external shapes. Subsequent HBC/OCS hydrogel implants showed good cell viability results for human adipose-derived mesenchymal stem cell (HAMSCs) cultures in vitro. Moreover, the hydrogels were shown to elicit the least amount of pro-inflammatory gene expression of macrophages and to inhibit acute immune responses [147]. It is reported by C. Luo et al. that a hybrid low-temperature extrusion-based 3D bioprinting was adopted to deposit cell-laden GelMA hydrogels for cartilage tissue engineering [148]. It was shown that at a concentration of 5% (*w/v*), the bioprintability (such as sol-gel transition and shear-thinning behavior) of GelMA hydrogels could be improved by changing the deposition temperature. These research findings highlight the path forward for 3D bioprinting of bone and cartilage tissue.

### 4.3. Other Tissue Engineering Applications

Skin is the first layer of protection for the human body from foreign substances. Many diseases caused by infection from damaged skin remain leading causes of death worldwide. It is critical to apply 3D bioprinting technology for the fabrication of skin substitutes for the repair of damaged skin. S. Michael et al. reported using the LAB technique to craft skin-like tissues of layers of NIH3T3 fibroblasts and HaCaT keratinocytes. The obtained crafts were transplanted into skin wounds of nude mice, and the attachment of the crafts to skin tissue and cell proliferation and differentiation were observed [149].

**Figure 7:**
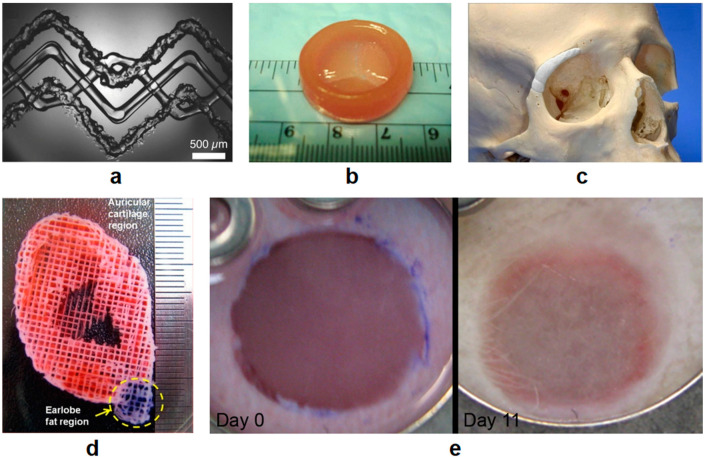
(**a**) Bright field microscopy image of a 3D-printed vascularized and heterogeneous tissue construct (reproduced with permission from J. A. Lewis et al., Advanced Materials; published by John Wiley and Sons, 2014) [133]. (**b**) 3D-printed heart valve conduit (reproduced with permission from B. Duan et al., Acta Biomaterialia; published by Elsevier, 2014) [134]. (**c**) 3D printed and inserted BCP implant with adequate fitting (reproduced with permission from M. Castilho et al., Biofabrication; published by IOP Publishing, 2014) [142]. (**d**) Acellular fabricated ear-shaped structure using 3D bioprinting technology with the sacrificial layer process, auricular cartilage region (red color) and lobe fat region (blue color) (reproduced with permission from J-S Lee et al., Biofabrication; published by IOP Publishing, 2014) [145]. (**e**) Tissue-engineered skin construct in the dorsal skin fold chamber in nude mice (the left picture shows the implantation directly inserted into the wound on day 0 and the right picture shows the results on day 11) (reproduced with open access from S. Michael et al., PLOS ONE; published by Public Library of Science, 2013) [149].

In general, patients with liver transplantation demands have two options: getting healthy livers from donors or wait for the regeneration of their own liver tissues. However, both options are limited due to the high demands and short supply of donors and the extremely long self-regeneration process of liver tissue. Under this circumstance, 3D bioprinting of liver tissue is particularly important for enabling more options for liver transplantation. Primary hepatocytes and stem cell-derived hepatocytes have been used as the bioink to print liver tissue [150]. A printed liver tissue containing both cell types can be sustained for some time; however, some cell activity and functionality are lost during the printing process. Specification of the liver, such as size and shape, could be achieved via 3D bioprinting during a liver resection operation [151]. With advances in bioprinting, new techniques that can maintain cell activity and functionality over a longer time have been developed [152]. Some examples of the above-mentioned applications are shown in Figure 7.

### 4.4. Drug Screening

3D bioprinting is a promising new approach to designing drug screening systems. Compared to conventional manual screening techniques, bioprinting could uniformly distribute cells onto a microdevice surfaces, which is highly desirable for testing and screening the interactions between cells and the tested drugs [153,154]. R. Chang et al. developed a pneumatically-driven, extrusion-based bioprinter to build a drug testing platform for the liver with alginate encapsulated immortalized hepatocytes. This system mimics the in vivo microenvironments of different mammalian tissues and can differentiate the drug metabolism capacity useful for screening efficacy and toxicity for the agent of interest [155]. Other studies show that it is possible to integrate cells that cause skin disease into biomaterials to construct skin tissue via 3D bioprinting. In this way, skin tissue printed with pathogenic cells could be used to examine the pathophysiologies of skin diseases [156]. Bioprinting could also be used for cell seeding during the manufacturing of organ-on-a-chip devices, which simulate paths of typical organ functions, for the investigation of potential drug effects on tissues [157].

## 5. Challenges and Future Prospects

3D bioprinting is a promising new approach for tissue engineering with the ability to fabricate specific constructs with desirable structural and mechanical properties, and to directly deposit living cells with demanding biological functions for the regenerative building of scaffolds, tissues, and organs; however, the challenges related to specific technical, material, and cellular aspects of the bioprinting processes remain critical for the future development of 3D bioprinting.

There are needs for increased printing resolution, speed, and compatibility for biomaterials from a technological perspective. The resolution limitation for inkjet and extrusion-based bioprinting is imposed by the physical confinement of the nozzles, usually above 50 μm [49]. Laser-assisted bioprinting has a higher resolution in comparison due to the micron-scale focal size of the light beam from the micromirror in the laser source [158,159], which makes it possible to fabricate complex 3D structures at a submicron resolution. However, resolution improvement to the nanoscale (subcellular or molecular level) is required for existing bioprinting technologies to better control the physical guidance provided by the microarchitecture and the heterogeneous distribution of functional biomolecules, such as growth factors and peptide ligands [49]. Besides economic and productivity reasons for shortening the printing time in large scale production for clinical use, there are situations wherein extended working time is to the disadvantage of sustaining bioink properties and functionalities, and the maintenance of partially printed structures are required. The introduction of parallel bioprinters with multi-heads printing features, and other printing process refinements, such as continuous liquid interface production (CLIP) of 3D structures [160], has effectively reduced the printing time overall. As for hybrid 3D bioprinting, efforts should be made to seek milder processing conditions for better integration of AM and CM with cell encapsulation during manufacturing, especially for approaches such as LFDM, LDM, and RFP.

On the other hand, the selection of materials remains a significant challenge for 3D bioprinting. As mentioned in the previous section, a suitable material for bioprinting should be biocompatible, printing compatible, and possess structural and mechanical properties to support and maintain cellular viability and function. Biomimetic materials, such as dECM bioinks, are used to mimic the microarchitectures for better cell and tissue regeneration, but often lack the mechanical strength and require supporting materials that are stronger but less bioactive, such as PCL [161]. The use of tunable bioinks is promising for incorporating other desirable features such as biodegradability and biocompatibility with mechanical properties [162,163,164]. The restricted number of printable biomaterials also applies to hybrid 3D bioprinting, which caused the situation in which only a few constructs fabricated by hybrid methods have been extensively studied in vitro and in vivo [67]. Bioprinting requires sources of cells to proliferate and differentiate with control quickly, show no negative immune responses to patients, reproduce all functions of the tissues and organs, and survive the printing process with adequate viability and maintained functionality. Recent advances in the application of small molecules to cell culture make it promising for more control over manipulating cell growth with signs of directed differentiation [165,166,167,168].

Besides challenges from technology and material sources, with the increasing availability of personalized 3D printing and personal 3D printers, there will be significant urges for the regulation and supervision of specific printed products. One of the current concerns and challenges is that the wide accessibility of 3D bioprinting could lead to unregulated do-it-yourself (DIY) home use for tissue fabrication. Another concern is that bioprinting could potentially be used for bioterrorism to develop a bioweapon that threatens other people’s lives [169]. Other concerns involve ethical and regulatory issues for 3D bioprinting-related human clinical trials because the nature of the 3D bioprinting treatment is highly explicitly customized and designed for a targeted patient and that patient only. How ethical is it to test a bioprinted organ using the patient’s own cells but on someone else first [170]? Or how efficient is it for the patient to serve as his/her own testing subject and how could regulations play a role in situations like this to protect the benefits of the patients and the medical treatment suppliers? It will take years to develop a fit-for-purpose regulatory framework or specific regulatory guidance for effectively governing 3D bioprinted tissues in a global environment. However, efforts could be made and have been made towards the improvement of local regulation of 3D bioprinting. The Ministry of Food and Drug Safety in South Korea and the Pharmaceuticals and Medical Devices Agency in Japan have provided some specific regulatory guidance loosely applicable to 3D bioprinting, even though parts of the guidance concern only 3D printing in general and regulate only academic research and marketing authorization of limited types of 3D bioprinted products [171]. It is clear that the establishment of management and regulation is crucial for the sustainable development of 3D bioprinting technology [135].

## 6. Conclusions

3D bioprinting has shown excellent tissue engineering capability with numerous applications for regenerative medicine, transplantation, and drug discovery. As an advanced fabrication technique for sophisticated 3D constructs with desirable biological, structural, and mechanical properties, improvements are still urged for technological enhancements and a broader range of material selection. Interestingly, the hybrid 3D bioprinting technologies combining additive and conventional manufacturing have shown promising improvements in printing resolution, constructs of greater mechanical strength, native-like biological microenvironments, and cell-related activities. Regulation and supervision of 3D bioprinting are also needed for sustainable future development. Even with the progress made, 3D bioprinting remains an emerging and growing technology with an incredible potential in manufacturing and healthcare strategies.

## Figures and Tables

**Table 1:** Comparison of 3D printing techniques.

	Methods	Advantages	Disadvantages	Characteristics	References
Non-biological 3D printing	Fused deposition modeling (FDM)	Low cost, fast and easy process	High processing temperature	Continuous filaments of thermoplastics are heated into a semi-liquid state for extrusion	[14,17] [20,21,22]
	Stereolithography (SLA)	Extremely high resolution, good for complex structures	Cytotoxicity, weak mechanical properties	UV light or electron beams to initiate polymerization reactions, nozzle-free	[33,34,35]
	Selective laser sintering (SLS)	Superior mechanical properties	Limited material selection, low resolution	Powder bed fusion process, high energy input, nozzle-free	[38,39]
	Electron beam melting (EBM)				
	Direct energy deposition (DED)	Bulk metal repair and retrofit			[4]
3D bioprinting	Inkjet bioprinting	Low cost, fast printing, widely accessible	Nozzle clogging	Conventional inkjet printing based technique	[47,48,49]
	Laser-assisted bioprinting (LAB)	Non-contact, high cell viability	Complex operation, time consuming preparation	“Ribbon” structure preparation needed for printing material, nozzle-free	[3,13] [57,58,59]
	Extrusion bioprinting	Deposition of high-density cells	Low cell viability	Continuous filaments of bioink extruded by various driving forces	[3,62]

**Table 2:** Summary of bioprinting materials.

	Advantages	Disadvantages	Advances	References
Natural polymers	Close to native ECM and inherent bioactivities	Limited tunability	dECM in bioinks to closely resemble natural tissues	[115,116]
Synthetic polymers	Adjustable chemical and mechanical properties	Poor biocompatibility	Pluronics as a sacrificial material with outstanding bioprintability	[117,118,119]
			PNIPAAM incorporated with other polymers for excellent printing resolution and high cell viability	[120]
Cells	Good proliferation and differentiation capability by STEM cells	Finite lifespan and culturing difficulties by many primary cell types	Stem cells from bone marrow and fat; perinatal stem cells from amniotic fluid	[125,126,127,128,129]
